# Supplementary material for: Sensory neuron lineage mapping and manipulation in the Drosophila olfactory system
Source: Nat Commun. 2019 Feb 7;10:643. doi: 10.1038/s41467-019-08345-4 (PMC6367400; doi:10.1038/s41467-019-08345-4)
Supplement: Supplementary file 1 — Supplementary Information [file 41467_2019_8345_MOESM1_ESM.pdf]

Supplementary Information

Sensory neuron lineage mapping and manipulation in the *Drosophila* olfactory system

Phing Chian Chai, Steeve Cruchet, Leonore Wigger and Richard Benton

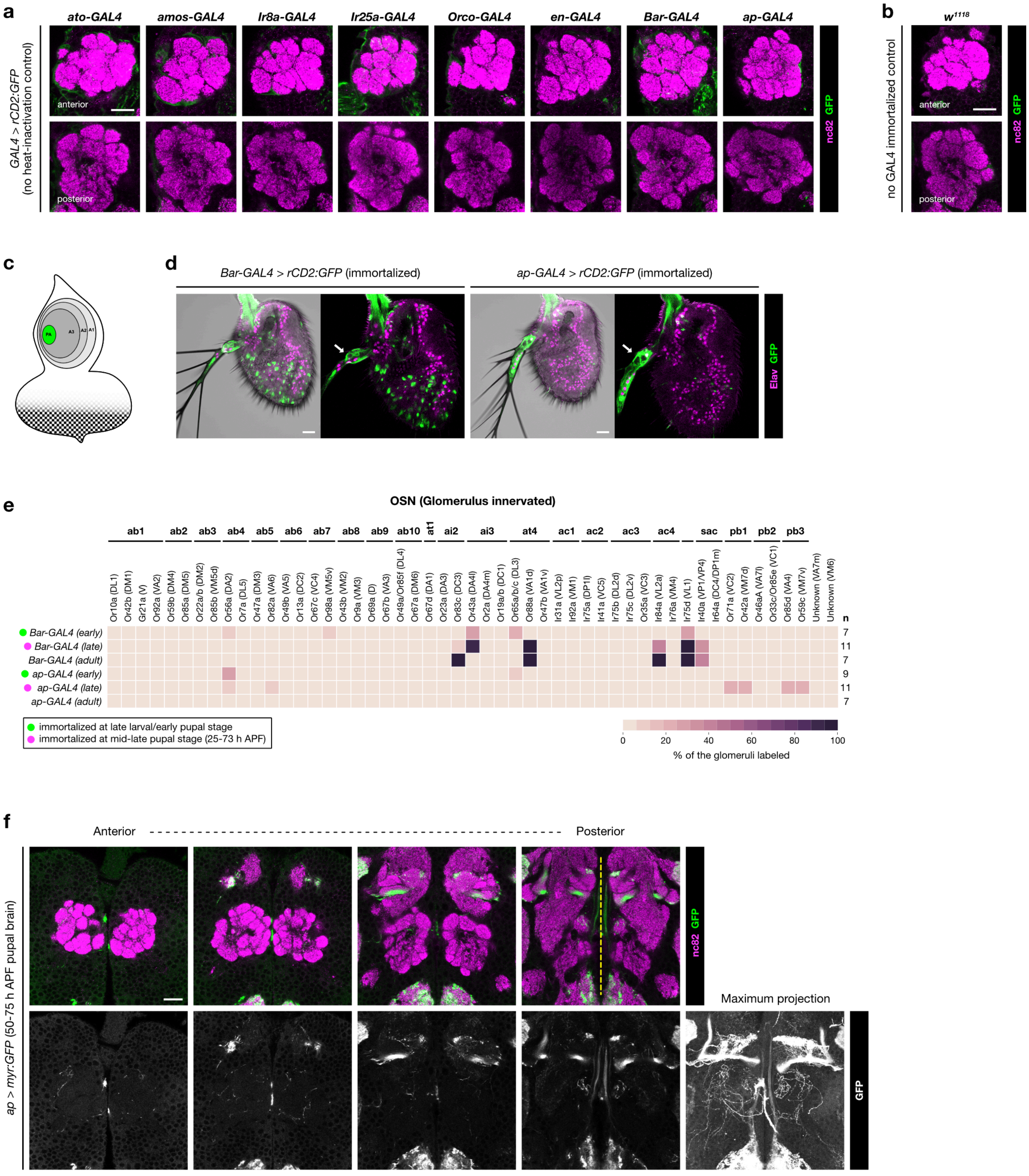

Supplementary Figure 1. Control experiments for the genetic immortalization system.

(a) "No heat-inactivation" controls for the immortalized *enhancer*-GAL4 system. Omitting the heat-inactivation step and culturing animals at 19ºC throughout development prevents rCD2:GFP expression (green) in all the OSN axons innervating the antennal lobe (glomeruli are visualized with nc82 (magenta)). Scale bar = 20 µm in this and other panels. The genotypes of the flies used in the figures are listed in Supplementary Table 1.

(b) A "no GAL4" control for the immortalization system. Substituting the *enhancer*-GAL4 with chromosomes from control animals (w^{1118}) abolishes rCD2:GFP

expression (green) in the OSN axons innervating the antennal lobe (glomeruli are visualized with nc82 (magenta))

(c) The aristal SOPs are located in the presumptive arista (PA) area (green) of the antennal disc.

(d) Immortalization of *Bar*-GAL4 and *ap*-GAL4 in a 4 h BPF-20 h APF window produced GFP labeled neurons (green) in aristal neurons (GFP signal elsewhere in the antenna is non-neuronal background).

(e) Heatmap showing the frequency that a given antennal lobe glomerulus is innervated by OSN axons labeled by a GFP reporter (either myr:GFP or rCD2:GFP) under the control of *Bar*-GAL4 or *ap*-GAL4. The immortalization of these *enhancer*-GAL4 lines was performed at two different time windows: early (9 h BPF-20 h APF) and late (25-73 h APF). The glomerular innervation frequencies of GFP-labeled OSN axons under non-immortalized conditions at the adult stage are also shown as a reference. The number of antennal lobes scored (n) is indicated on the right of the heatmap.

(f) Anteroposterior sections and a maximum projection of antennal lobes in which the glomeruli are labeled by nc82 (magenta) and neurons expressing *ap*-GAL4 are marked by a myr:GFP reporter (green). The labeled neurons that innervate the antennal lobe (previously interpreted to be OSNs[1]) lack dorsal commissures – which should cross the midline (dashed yellow line) – and appear to terminate in multiple glomeruli, indicating that they are unlikely to be antennal-derived OSNs.

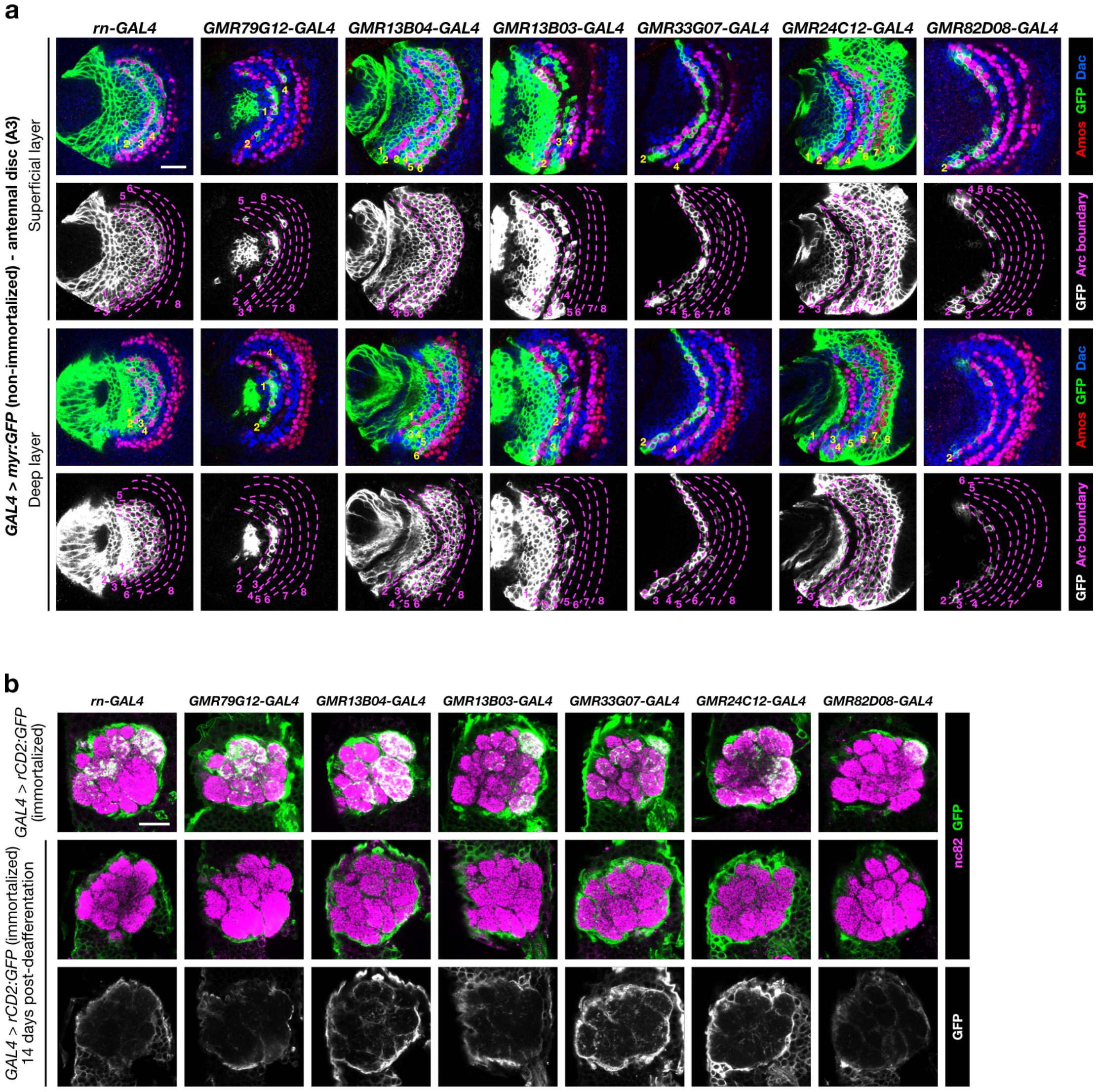

Supplementary Figure 2. The fate mapping is free from interference from non-olfactory sensory neurons.

(a) Rows 1 and 3: non-immortalized *enhancer*-GAL4 driven expression of myr:GFP (green), colabeled with α-Amos (red) and α-Dac (blue) in 2 h APF antennal discs (the same confocal images were shown in Fig. 2d).
Rows 2 and 4: the GFP channel from the corresponding panels in rows 1 and 3 are shown in grey scale to highlight the degree of *enhancer*-GAL4 expression in each arc, which are demarcated by virtual boundaries (dashed magenta lines).

(b) Row 1: immortalized *enhancer*-GAL4 driven expression of rCD2:GFP (green) in OSN axons innervating different subsets of glomeruli labeled by nc82 (magenta). Rows 2-3: immortalized *enhancer*-GAL4 driven expression of rCD2:GFP is lost in the antennal lobe glomeruli 14 days after the antennae were surgically removed. Scale bar = 20 μm.

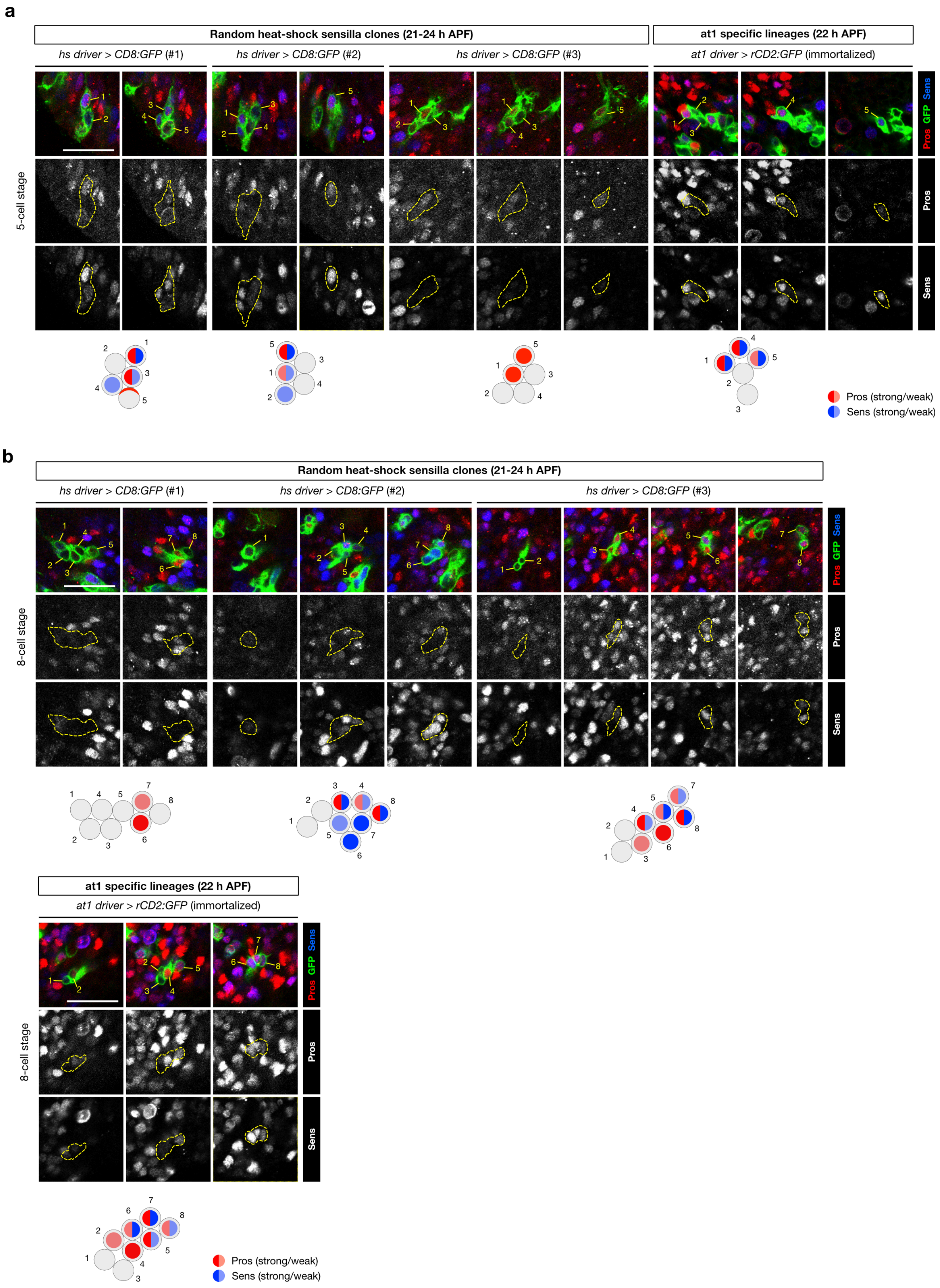
a
Random heat-shock sensilla clones (21-24 h APF)
hs driver > CD8:GFP (#1)
hs driver > CD8:GFP (#2)
hs driver > CD8:GFP (#3)
at1 specific lineages (22 h APF)
at1 driver > rCD2:GFP (immortalized)
5-cell stage
Pros GFP Sens
Pros
Sens
Pros (strong/weak)
Sens (strong/weak)
b
Random heat-shock sensilla clones (21-24 h APF)
hs driver > CD8:GFP (#1)
hs driver > CD8:GFP (#2)
hs driver > CD8:GFP (#3)
8-cell stage
Pros GFP Sens
Pros
Sens
at1 specific lineages (22 h APF)
at1 driver > rCD2:GFP (immortalized)
8-cell stage
Pros (strong/weak)
Sens (strong/weak)

c

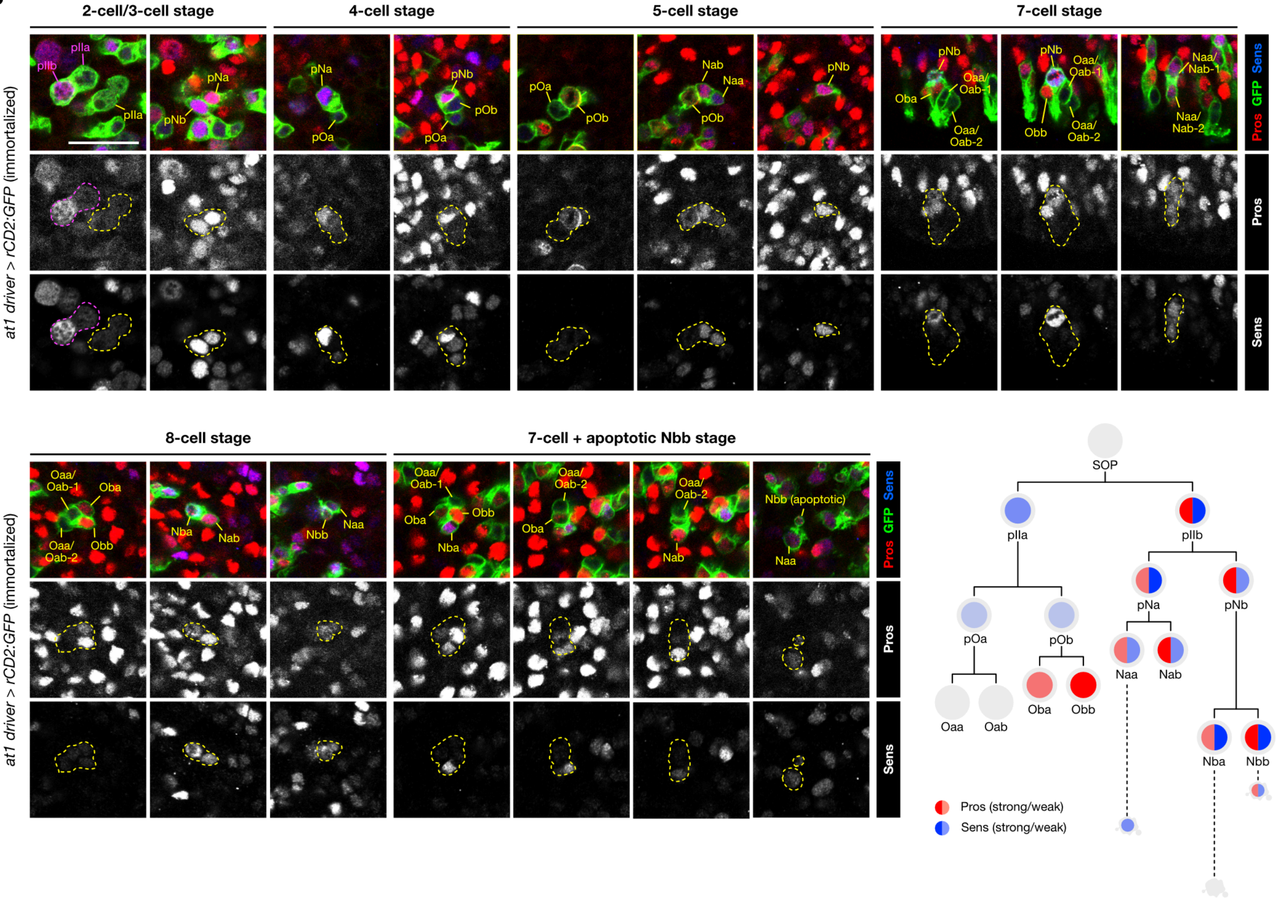

2-cell/3-cell stage
4-cell stage
5-cell stage
7-cell stage
8-cell stage
7-cell + apoptotic Nbb stage
at1 driver > rCD2:GFP (immortalized)
Pros GFP Sens
Pros
Sens
pIIb
pIIa
pNa
pNb
pOa
pOb
Nab
Naa
Oba
Oaa/ Oab-1
Oaa/ Oab-2
Obb
Naa/ Nab-1
Naa/ Nab-2
Nba
Nbb (apoptotic)
SOP
Oaa
Oab
Nbb
Pros (strong/weak)
Sens (strong/weak)

d

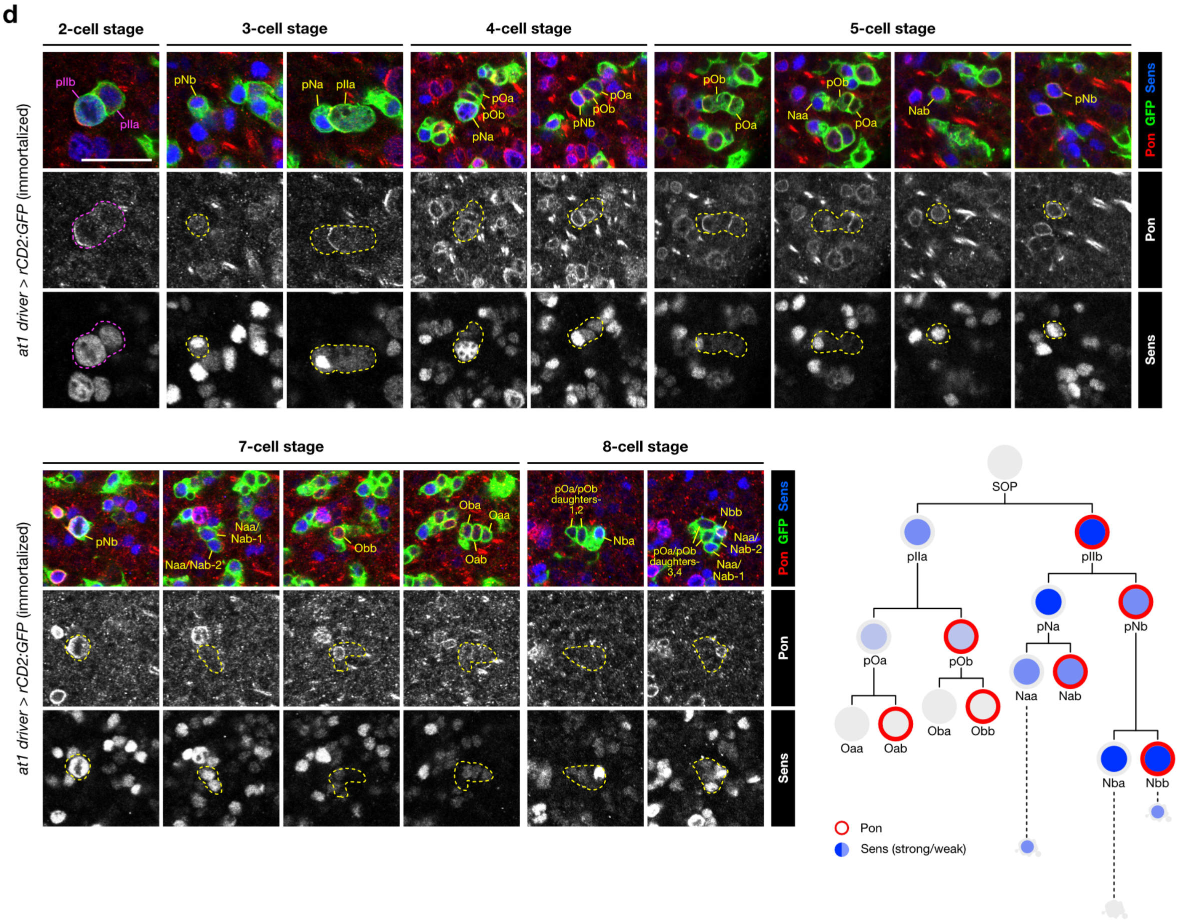

2-cell stage
3-cell stage
4-cell stage
5-cell stage
7-cell stage
8-cell stage
at1 driver > rCD2:GFP (immortalized)
Pon GFP Sens
Pon
Sens
pIIb
pIIa
pNb
pNa
pOa
pOb
Naa
Nab
Naa/ Nab-1
Naa/Nab-2
Obb
Oba
Oaa
Oab
pOa/pOb daughters-1,2
Nba
Nbb
pOa/pOb daughters-3,4
Naa/ Nab-2
Naa/ Nab-1
SOP
Pon
Sens (strong/weak)

e

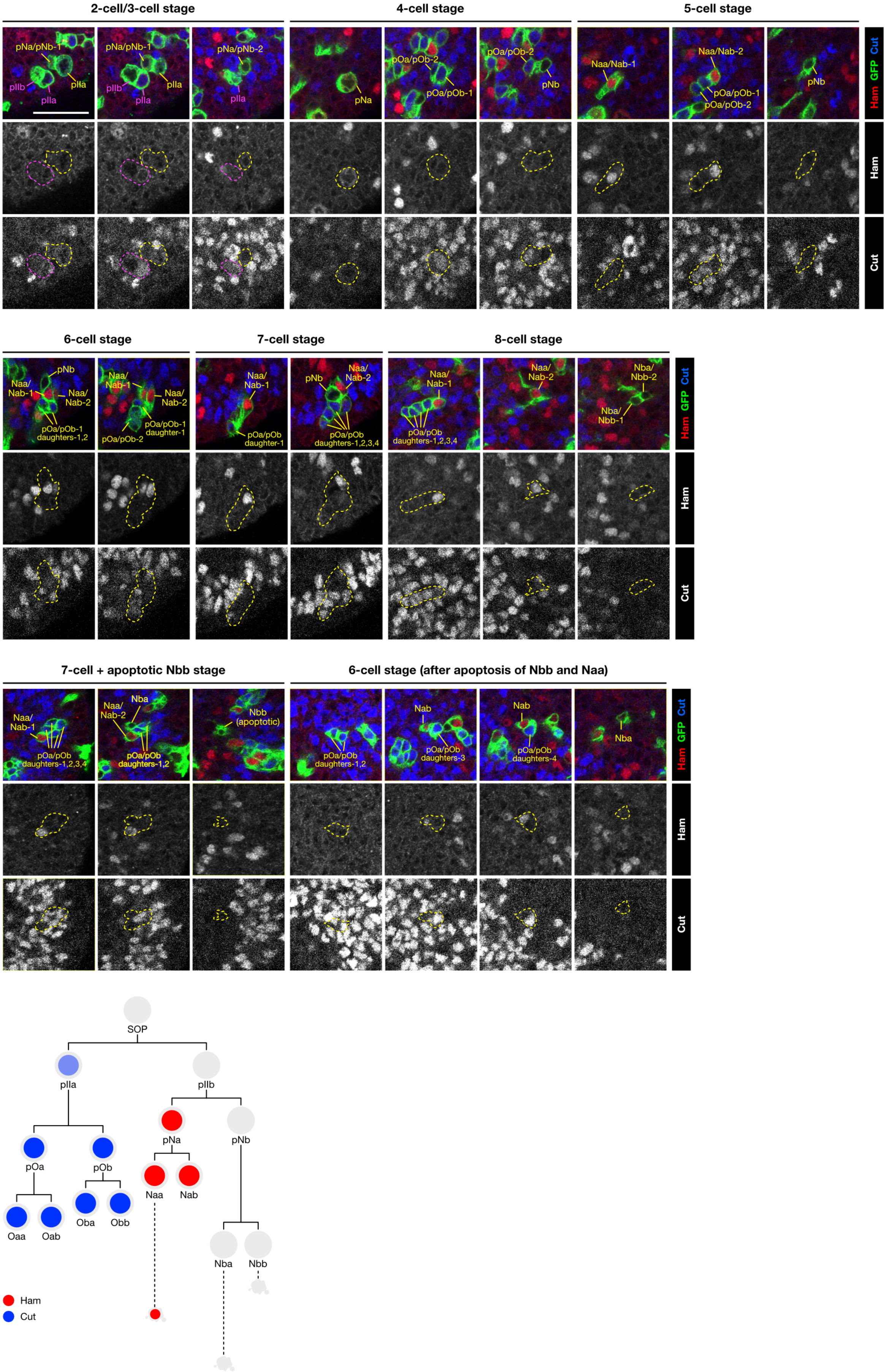

2-cell/3-cell stage
4-cell stage
5-cell stage
6-cell stage
7-cell stage
8-cell stage
7-cell + apoptotic Nbb stage
6-cell stage (after apoptosis of Nbb and Naa)
at1 driver > rCD2:GFP (immortalized)
Ham GFP Cut
Ham
Cut
pNa/pNb-1
pNa/pNb-2
pIIb
pIIa
pNa
pNb
pOa/pOb-1
pOa/pOb-2
Naa/Nab-1
Naa/Nab-2
pOa/pOb-1 daughters-1,2
pOa/pOb-1 daughter-1
pOa/pOb daughter-1
pOa/pOb daughters-1,2,3,4
Nba/Nbb-1
Nba/Nbb-2
Nba
Nbb (apoptotic)
pOa/pOb daughters-1,2
pOa/pOb daughters-3
pOa/pOb daughters-4
Nab
SOP
pOa
pOb
Oaa
Oab
Oba
Obb
Naa
Nbb
Ham
Cut

Supplementary Figure 3. at1 lineages show invariant molecular marker expression.

(a) Left: the 5-cell stage of random SOP lineages (visualized by heat shock-based clonal GFP labeling (green); clonal generation time: 48-64 h ALH) in developing pupal antennae at 21-24 h APF. The three lineages shown have distinct expression profiles for Pros (red) and Sens (blue). Right: by contrast, a 5-cell stage of the at1 lineage (visualized with the immortalized at1 driver) has highly predictable Pros and Sens expression profiles. Schematic summaries of the expression patterns are shown below the images. Scale bar = 20 μm in this and other panels.

(b) Top: the 8-cell stage of random SOP lineages (visualized by heat shock-based clonal GFP labeling as in (a)) in 21-24 h APF antennae have highly variable Pros (red) and Sens (blue) expression patterns. Bottom: by contrast, the 8-cell stage of the at1 lineage shows invariant Pros and Sens expression profiles.

(c) – (e) Expression profiles of (c) Pros (red), Sens (blue), (d) Pon (red), Sens (blue), and (e) Ham (red), Cut (blue), in at1 lineages visualized with the immortalized at1 driver (green). Schematics summarizing the expression profiles are shown after each image series.

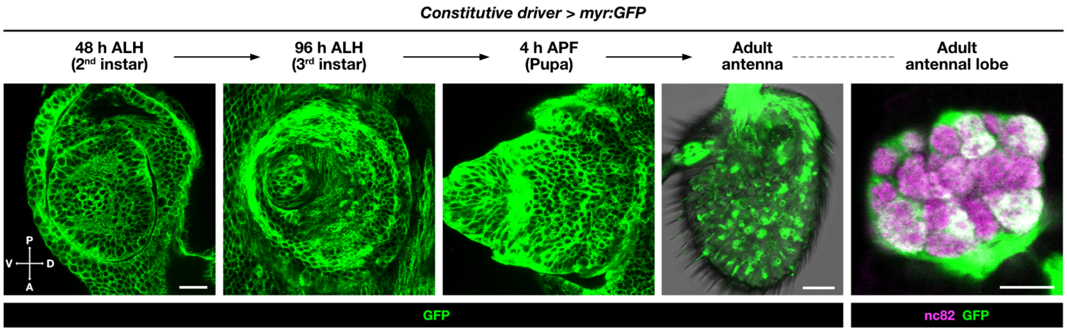

Supplementary Figure 4. Expression analysis of the constitutive GAL4 driver. Constitutive GAL4-driven expression of membrane myr:GFP (green) is found in all cells within the developing antennal disc at 48 h after larval hatching (ALH) (2nd instar), 96 h ALH (3rd instar) and 4 h APF (pupa). This expression persists in the progeny of these cells in the adult antenna (overlaid on a brightfield background), including in the OSNs whose axons innervate the adult antennal lobe (visualized with nc82; magenta). Scale bars = 20 µm.

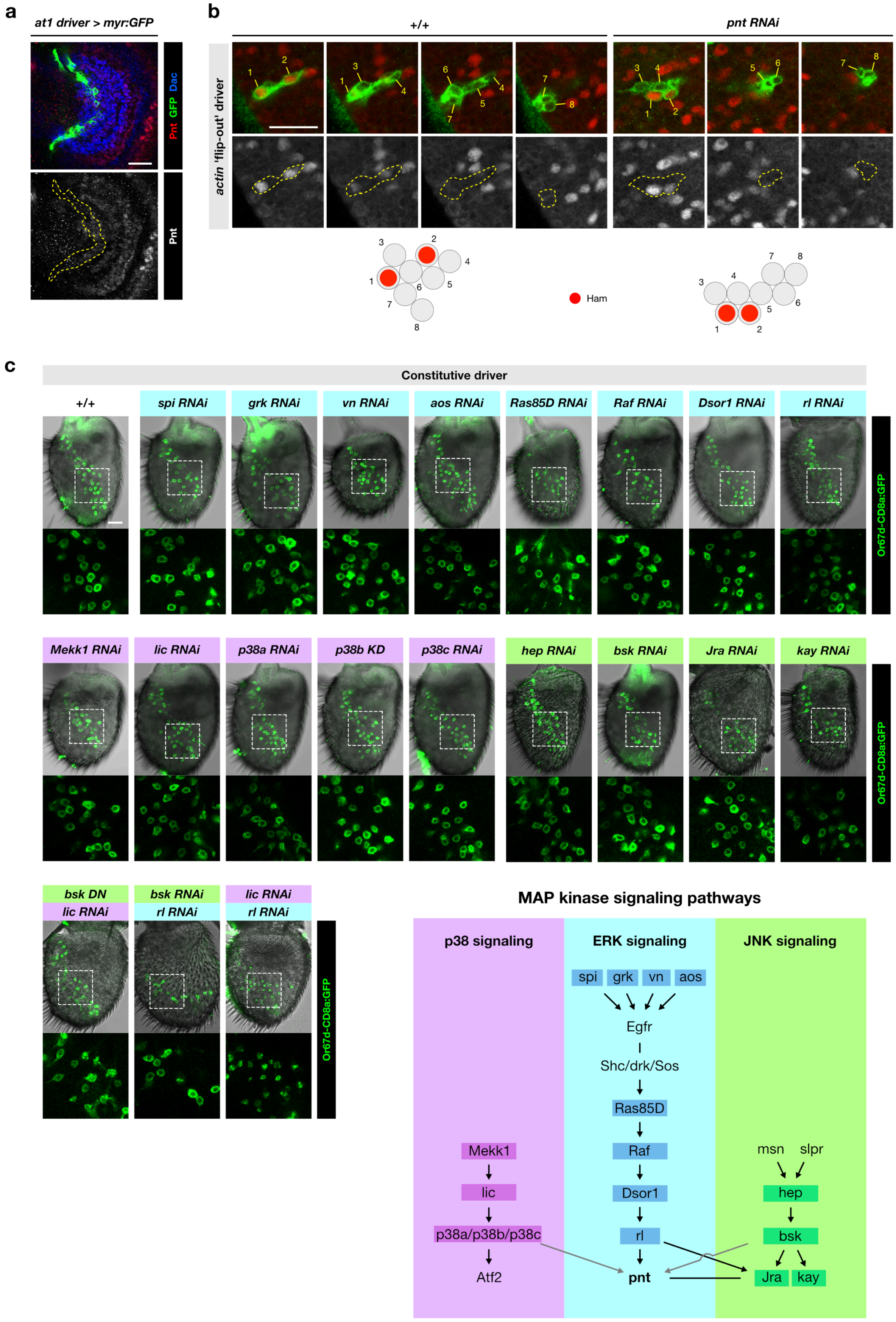
a
at1 driver > myr:GFP
Pnt GFP Dac
Pnt
b
+/+
pnt RNAi
actin 'flip-out' driver
Ham
c
Constitutive driver
+/+
spi RNAi
grk RNAi
vn RNAi
aos RNAi
Ras85D RNAi
Raf RNAi
Dsor1 RNAi
rl RNAi
Or67d-CD8a:GFP
Mekk1 RNAi
lic RNAi
p38a RNAi
p38b KD
p38c RNAi
hep RNAi
bsk RNAi
Jra RNAi
kay RNAi
Or67d-CD8a:GFP
bsk DN
lic RNAi
bsk RNAi
rl RNAi
lic RNAi
rl RNAi
Or67d-CD8a:GFP
MAP kinase signaling pathways
p38 signaling
ERK signaling
JNK signaling
spi
grk
vn
aos
Egfr
Shc/drk/Sos
Ras85D
Raf
Dsor1
rl
pnt
Mekk1
lic
p38a/p38b/p38c
Atf2
msn
slpr
hep
bsk
Jra
kay

Supplementary Figure 5. The role of Pnt in Naa fate specification is unrelated to MAP kinase signaling pathways.

(a) Pnt (red) is expressed at low levels in the at1 SOPs (green) of a 2 h APF antennal disc; Dac (blue) demarcates the A3 region. Scale bar = 20 µm in this and other panels.

(b) Left: representative control 8-cell heat-shock clone (green) revealing two cells expressing Ham (red). Right: in a representative 8-cell *pnt* RNAi clone (green), Ham expression is unaffected. Schematics summarizing Ham expression in these clones are shown below the images.

(c) Antennae expressing the *Or67d*-CD8a:GFP reporter (green, overlaid on a brightfield background) in control animals or with single or double RNAi (using the constitutive driver) of genes in three main MAPK signaling pathways. For two experiments, non-RNAi-based tools were used: *bsk* dominant negative (DN) and *p38b* KD (kinase dead). The areas within the dashed boxes are magnified to show the cell bodies of GFP-expressing neurons; no doublets are observed, in contrast to *pnt* RNAi antennae (Fig. 7a). Bottom right: schematic of the core components of p38 (purple), ERK (cyan) and JNK (green) signaling pathways. The black arrows/lines indicate known interactions in *Drosophila*, while the grey arrows indicate interactions reported for their mammalian homologs.

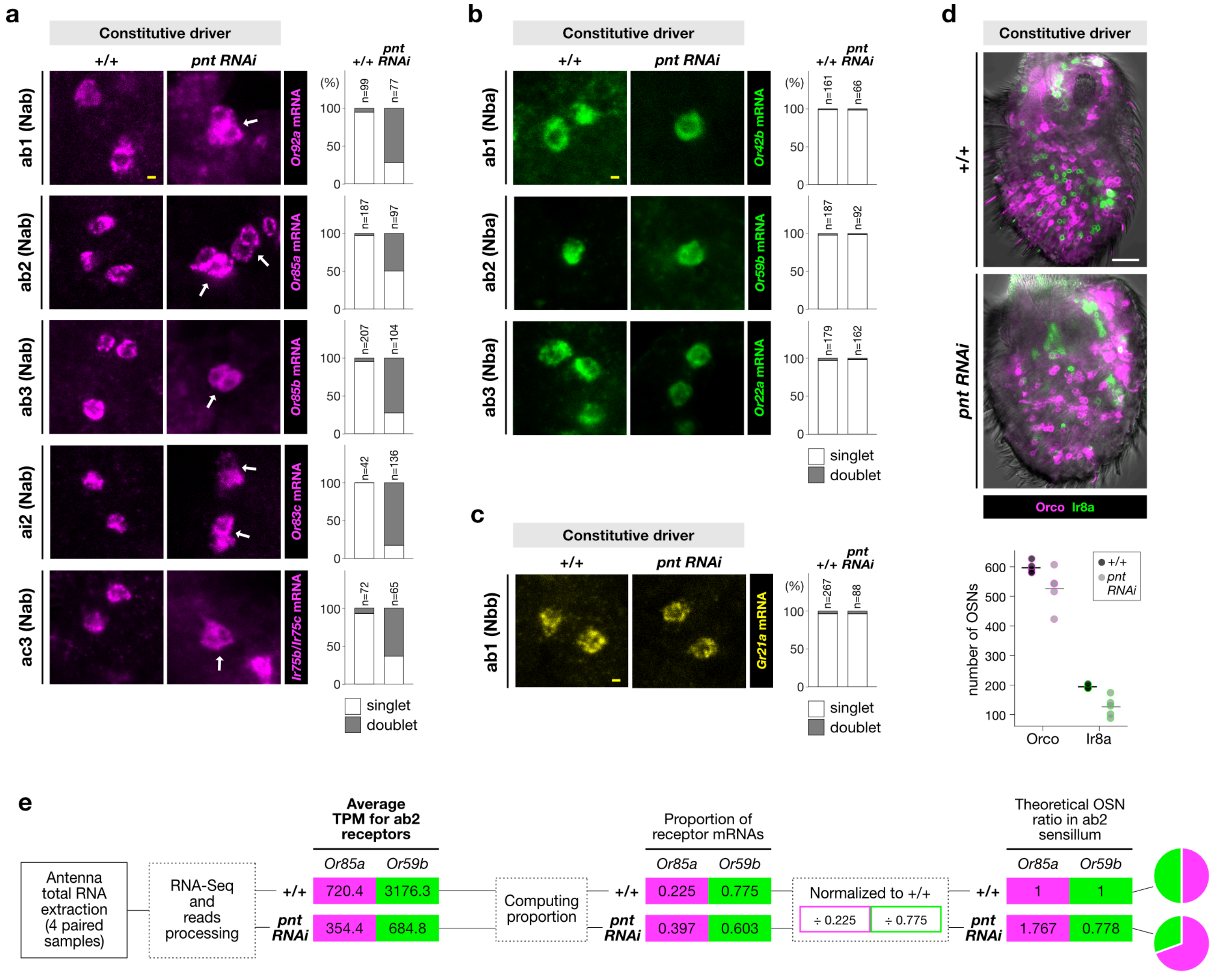

Supplementary Figure 6. Loss of Pnt results in Naa-to-Nab transformations in diverse sensillar subtypes, and a global reduction in sensilla number.

(a) OSNs of Nab fate, labeled by FISH probes against their olfactory receptor mRNAs (magenta), in the indicated sensillar classes in control and *pnt* RNAi antennae. *pnt* RNAi antennae exhibit doublets of Nab, although phenotypic penetrance was variable between different sensillar subtypes, possibly due to the contribution of other neural fate determinants. Yellow scale bar = 2 µm in this and other panels.

(b) OSNs of Nba fate, labeled by FISH probes against their olfactory receptor mRNAs (green), in the indicated sensillar classes in control and *pnt* RNAi antennae.

(c) The OSN of Naa fate in ab1 sensilla, labeled by a FISH probe against *Gr21a* mRNA (yellow), in control and *pnt* RNAi antennae.

(d) Immunolabeling with α-Orco (magenta) and α-Ir8a (green) – which together cover most antennal OSNs – in control and *pnt* RNAi antennae; fluorescent signals are overlaid on a brightfield background. The numbers of Orco- and Ir8a-expressing OSNs are reduced upon loss of *pnt*: the distributions of the OSN counts are presented as strip-plots (horizontal line denotes the sample mean; n=5). Mann-Whitney U test: p=0.047 and p=0.006 for Orco- and Ir8a-expressing OSNs, respectively, comparing control and *pnt* RNAi genotypes. White scale bar = 20 µm.

(e) Workflow to infer OSN numbers in sensilla in *pnt* RNAi antennae (Fig. 9f) using, as an example, the read counts for ab2 receptor transcripts (*Or85a* and *Or59b*). TPM: transcripts per million.

Supplementary Table 1. Genotypes of the experimental animals.

Figure Panels	Genotypes
Fig. 1d (Rows 1-3), Fig. 1e	(i) *ato*^{NP6558}*/10xUAS-myr:GFP*<br>(ii) *amos-GAL4/+; 10xUAS-myr:GFP/+*<br>(iii) *Ir8a-GAL4/+; 10xUAS-myr:GFP/+*<br>(iv) *Ir25a-GAL4/10xUAS-myr:GFP*<br>(v) *Orco-GAL4/+; 10xUAS-myr:GFP/+*<br>(vi) *en2.4-GAL4/+; 10xUAS-myr:GFP/+*<br>(vii) *Bar*^{NP4099}*/+; 10xUAS-myr:GFP/+*<br>(viii) *ap*^{md544}*/+; 10xUAS-myr:GFP/+*
Fig. 1d (Rows 4-5), Fig. 1e Supplementary Fig. 1a	(i) *UAS-FLP, tub-GAL80*^{ts}*/+; ato*^{NP6558}*/lexAop-rCD2:GFP, act>STOP>*LHV_2<br>(ii) *amos-GAL4/UAS-FLP, tub-GAL80*^{ts}*; lexAop-rCD2:GFP, act>STOP>*LHV_2*/+*<br>(iii) *Ir8a-GAL4/UAS-FLP, tub-GAL80*^{ts}*; lexAop-rCD2:GFP, act>STOP>*LHV_2*/+*<br>(iv) *UAS-FLP, tub-GAL80*^{ts}*/+; Ir25a-GAL4/lexAop-rCD2:GFP, act>STOP>*LHV_2<br>(v) *Orco-GAL4/UAS-FLP, tub-GAL80*^{ts}*; lexAop-rCD2:GFP, act>STOP>*LHV_2*/+*<br>(vi) *en2.4-GAL4/UAS-FLP, tub-GAL80*^{ts}*; lexAop-rCD2:GFP, act>STOP>*LHV_2*/+*<br>(vii) *Bar*^{NP4099}*/+; UAS-FLP, tub-GAL80*^{ts}*/+; lexAop-rCD2:GFP, act>STOP>*LHV_2*/+*<br>(viii) *ap*^{md544}*/UAS-FLP, tub-GAL80*^{ts}*; lexAop-rCD2:GFP, act>STOP>*LHV_2*/+*
Supplementary Fig. 1b	(i) *UAS-FLP, tub-GAL80*^{ts}*/+; lexAop-rCD2:GFP, act>STOP>*LHV_2*/+*
Supplementary Fig. 1d	(i) *Bar*^{NP4099}*/+; UAS-FLP, tub-GAL80*^{ts}*/+; lexAop-rCD2:GFP, act>STOP>*LHV_2*/+*<br>(ii) *ap*^{md544}*/UAS-FLP, tub-GAL80*^{ts}*; lexAop-rCD2:GFP, act>STOP>*LHV_2*/+*
Supplementary Fig. 1e	(i) *Bar*^{NP4099}*/+; UAS-FLP, tub-GAL80*^{ts}*/+; lexAop-rCD2:GFP, act>STOP>*LHV_2*/+*<br>(ii) *ap*^{md544}*/UAS-FLP, tub-GAL80*^{ts}*; lexAop-rCD2:GFP, act>STOP>*LHV_2*/+*
Supplementary Fig. 1f	*ap*^{md544}*/+; 10xUAS-myr:GFP/+*
Fig. 2d (row 1-2), Supplementary Fig. 2a	*enhancer-GAL4/10xUAS-myr:GFP*<br>*enhancer-GAL4*: (i) *rn*^{89}*-GAL4* (ii) *GMR79G12-GAL4* (iii) *GMR13B04-GAL4* (iv) *GMR13B03-GAL4* (v) *GMR33G07-GAL4* (vi) *GMR24C12-GAL4* (vii) *GMR82D08-GAL4*
Fig. 2d (row 3-4), Supplementary Fig. 2b	*UAS-FLP, tub-GAL80*^{ts}*/+; enhancer-GAL4/lexAop-rCD2:GFP, act>STOP>*LHV_2<br>*enhancer-GAL4*: (i) *rn*^{89}*-GAL4* (ii) *GMR79G12-GAL4* (iii) *GMR13B04-GAL4* (iv) *GMR13B03-GAL4* (v) *GMR33G07-GAL4* (vi) *GMR24C12-GAL4* (vii) *GMR82D08-GAL4*
Fig. 4a, Supplementary Fig. 5a	*GMR82D08-GAL4/10xUAS-myr:GFP*
Fig. 4b-d, Fig. 5a-b, Supplementary Fig. 3c-e, Fig. 8a	*UAS-FLP, tub-GAL80*^{ts}*/+; GMR82D08-GAL4/lexAop-rCD2:GFP, act>STOP>*LHV_2
Supplementary Fig. 3a-b	(i) *hs-FLP, UAS-mCD8:GFP/+; AyGAL4/+*<br>(ii) *UAS-FLP, tub-GAL80*^{ts}*/+; GMR82D08-GAL4/lexAop-rCD2:GFP, act>STOP>*LHV_2
Supplementary Fig. 4	*hs-FLP, UAS-mCD8:GFP/+; AyGAL4/10xUAS-myr:GFP*
Fig. 6c	(i) *ey-FLP, Or67d-mCD8a:GFP/+; act5c(FRT-CD2)-GAL4/+*

	(ii) *ey-FLP, Or67d-mCD8a:GFP/UAS-Eip93F$^{RNAi-KK108140}$; act5c(FRT-CD2)-GAL4/+*<br>(iii) *ey-FLP, Or67d-mCD8a:GFP/+; act5c(FRT-CD2)-GAL4/UAS-lilli$^{RNAi-TRiP.HMS01066}$*<br>(iv) *ey-FLP, Or67d-mCD8a:GFP/+; act5c(FRT-CD2)-GAL4/UAS-CG15514$^{RNAi-TRiP.JF02231}$*<br>(v) *ey-FLP, Or67d-mCD8a:GFP/UAS-pnt$^{RNAi-KK100473}$; act5c(FRT-CD2)-GAL4/+*<br>(vi) *ey-FLP, Or67d-mCD8a:GFP/+; act5c(FRT-CD2)-GAL4/UAS-CG18262$^{RNAi-TRiP.HMS00215}$*<br>(vii) *ey-FLP, Or67d-mCD8a:GFP/UAS-Nf-YC$^{RNAi-TRiP.HMJ22254}$; act5c(FRT-CD2)-GAL4/+*<br>*ey-FLP, Or67d-mCD8a:GFP/+; act5c(FRT-CD2)-GAL4/UAS-ash1$^{RNAi-TRiP.JF01498}$*
Fig. 7a	(i) *ey-FLP, Or67d-mCD8a:GFP/+; act5c(FRT-CD2)-GAL4/+*<br>(ii) *ey-FLP, Or67d-mCD8a:GFP/UAS-pnt$^{RNAi-KK100473}$; act5c(FRT-CD2)-GAL4/+*<br>(iii) *ey-FLP, Or67d-mCD8a:GFP/+; act5c(FRT-CD2)-GAL4/UAS-pnt$^{RNAi-TRiP.JF02227}$*<br>(iv) *peb-GAL4/+; Or67d-mCD8a:GFP/+*<br>(v) *peb-GAL4/+; Or67d-mCD8a:GFP/UAS-pnt$^{RNAi-KK100473}$*
Fig. 7b	(i) *Or67d-mCD8a:GFP/UAS-pnt$^{RNAi-KK100473}$; GMR82D08-GAL4/GMR82D08-GAL4*<br>(ii) *Or67d-mCD8a:GFP/UAS-pnt$^{RNAi-KK100473}$; GMR82D08-GAL4/UAS-GAL4*
Fig. 7c	(i) *ey-FLP, Or67d-mCD8a:GFP/+; act5c(FRT-CD2)-GAL4/+*<br>(ii) *ey-FLP, Or67d-mCD8a:GFP/UAS-pnt$^{RNAi-KK100473}$; act5c(FRT-CD2)-GAL4/+*
Fig. 7d	(i) *hs-FLP, elav-GAL4/+; UAS-mCD8:GFP/+; FRT82B/FRT82B, tub-GAL80*<br>(ii) *hs-FLP, elav-GAL4/+; UAS-mCD8:GFP/+; FRT82B, pnt$^{\Delta 88}$/FRT82B, tub-GAL80*
Fig. 8c, Supplementary Fig. 5b	(i) *hs-FLP, UAS-mCD8:GFP/+; AyGAL4/+*<br>(ii) *hs-FLP, UAS-mCD8:GFP/+; AyGAL4/UAS-pnt$^{RNAi-KK100473}$*
Fig. 8d	*hs-FLP, UAS-mCD8:GFP/+; AyGAL4/+; UAS-pnt.P1/+*
Fig. 8e	*ey-FLP, Or67d-mCD8a:GFP/+; act5c(FRT-CD2)-GAL4/+; UAS-svp$^{RNAi-TRiP.JF03105}$/+*
Fig. 8f	(i) *ey-FLP, Or67d-mCD8a:GFP/+; act5c(FRT-CD2)-GAL4/+; UAS-svp.II /+*<br>(ii) *ey-FLP, Or67d-mCD8a:GFP/UAS-svp1; act5c(FRT-CD2)-GAL4/+* (not shown)<br>(iii) *ey-FLP, Or67d-mCD8a:GFP/UAS-pnt$^{RNAi-KK100473}$; act5c(FRT-CD2)-GAL4/+*<br>(iv) *ey-FLP, Or67d-mCD8a:GFP/UAS-pnt$^{RNAi-KK100473}$; act5c(FRT-CD2)-GAL4/UAS-svp.II*
Supplementary Fig. 5c	(i) *ey-FLP, Or67d-mCD8a:GFP/+; act5c(FRT-CD2)-GAL4/+*<br>(ii) *ey-FLP, Or67d-mCD8a:GFP/+; act5c(FRT-CD2)-GAL4/UAS-spi$^{RNAi-TRiP.JF03322}$*<br>(iii) *ey-FLP, Or67d-mCD8a:GFP/UAS-grk$^{RNAi-TRiP.HMC04213}$; act5c(FRT-CD2)-GAL4/+*<br>(iv) *ey-FLP, Or67d-mCD8a:GFP/UAS-vn$^{RNAi-TRiP.HMC04390}$; act5c(FRT-CD2)-GAL4/+*<br>(v) *ey-FLP, Or67d-mCD8a:GFP/+; act5c(FRT-CD2)-GAL4/UAS-aos$^{RNAi-TRiP.JF03020}$*<br>(vi) *ey-FLP, Or67d-mCD8a:GFP/+; act5c(FRT-CD2)-GAL4/UAS-Ras85D$^{RNAi-TRiP.JF02478}$*<br>(vii) *ey-FLP, Or67d-mCD8a:GFP/+; act5c(FRT-CD2)-GAL4/UAS-Raf$^{RNAi-TRiP.HMC04133}$*<br>(viii) *ey-FLP, Or67d-mCD8a:GFP/+; act5c(FRT-CD2)-GAL4/UAS-Dsor1$^{RNAi-TRiP.JF03100}$*<br>(ix) *ey-FLP, Or67d-mCD8a:GFP/+; act5c(FRT-CD2)-GAL4/UAS-rl^{RNAi-}*

	$^{\text{TRiP.HMS00173}}$<br>(x) *ey-FLP, Or67d-mCD8a:GFP/+; act5c(FRT-CD2)-GAL4/UAS-Mekk1*$^{\text{RNAi-TRiP.HM05075}}$<br>(xi) *ey-FLP, Or67d-mCD8a:GFP/UAS-lic*$^{\text{RNAi-TRiP.HMS05002}}$*; act5c(FRT-CD2)-GAL4/+*<br>(xii) *ey-FLP, Or67d-mCD8a:GFP/+; act5c(FRT-CD2)-GAL4/UAS-p38a*$^{\text{RNAi-TRiP.HMS01224}}$<br>(xiii) *ey-FLP, Or67d-mCD8a:GFP/UAS-p38b*^{KD}*; act5c(FRT-CD2)-GAL4/+*<br>(xiv) *ey-FLP, Or67d-mCD8a:GFP/+; act5c(FRT-CD2)-GAL4/UAS-p38c*$^{\text{RNAi-TRiP.HMC05719}}$<br>(xv) *ey-FLP, Or67d-mCD8a:GFP/+; act5c(FRT-CD2)-GAL4/UAS-hep*$^{\text{RNAi-TRiP.JF03137}}$<br>(xvi) *ey-FLP, Or67d-mCD8a:GFP/+; act5c(FRT-CD2)-GAL4/UAS-bsk*$^{\text{RNAi-TRiP.HMS00777}}$<br>(xvii) *ey-FLP, Or67d-mCD8a:GFP/+; act5c(FRT-CD2)-GAL4/UAS-Jra*$^{\text{RNAi-TRiP.JF01184}}$<br>(xviii) *ey-FLP, Or67d-mCD8a:GFP/+; act5c(FRT-CD2)-GAL4/UAS-kay*$^{\text{RNAi-TRiP.HMS00254}}$<br>(xix) *UAS-bsk.DN/+; ey-FLP, Or67d-mCD8a:GFP/UAS-lic*$^{\text{RNAi-TRiP.HMS05002}}$*; act5c(FRT-CD2)-GAL4/+*<br>(xx) *ey-FLP, Or67d-mCD8a:GFP/UAS-bsk*$^{\text{RNAi-GD10555}}$*; act5c(FRT-CD2)-GAL4/UAS-rl*$^{\text{RNAi-TRiP.HMS00173}}$<br>(xxi) *ey-FLP, Or67d-mCD8a:GFP/UAS-lic*$^{\text{RNAi-TRiP.HMS05002}}$*; act5c(FRT-CD2)-GAL4/UAS-rl*$^{\text{RNAi-TRiP.HMS00173}}$
Fig. 9b-f, Supplementary Fig. 6a-e	(i) *ey-FLP, act5c(FRT-CD2)-GAL4/+*<br>(ii) *UAS-pnt*$^{\text{RNAi-KK100473}}$*/+; ey-FLP, act5c(FRT-CD2)-GAL4/+*

Supplementary Table 2. *Drosophila* stocks.

Stock	Source
10xUAS-myr:GFP	BL32197
act5c(FRT-CD2)-GAL4	BL4780
amos-GAL4	[2]
ap^{md544}	BL3041
ato^{NP6558}	DGRC105-257
AyGAL4 (act5c>y+>GAL4)	BL3953
Bar^{NP4099}	DGRC104-601
en2.4-GAL4	BL30564
ey-FLP	BL5576, BL5577
FRT82B, tub-GAL80	BL5135
GMR13B03-GAL4	BL46142
GMR13B04-GAL4	BL45034
GMR24C12-GAL4	BL49076
GMR33G07-GAL4	BL49757
GMR79G12-GAL4	BL40051
GMR82D08-GAL4	BL46724
hs-FLP, elav-GAL4	BL5145
hs-FLP, UAS-mCD8:GFP	BL28832
Ir25a-GAL4	[3] (chromosome III)
Ir8a-GAL4	BL41731
lexAop-rCD2:GFP	[4]
Or67d-mCD8a:GFP	[5]
Orco-GAL4	[6]
peb-GAL4	[7]
pnt$^{\Delta 88}$	BL861
rn^{89}*-GAL4*	[8]
UAS-aos$^{\text{RNAi-TRiP.JF03020}}$	BL28383
UAS-ash1$^{\text{RNAi-TRiP.JF03430}}$	BL36130
UAS-Br140$^{\text{RNAi-GD4038}}$	VDRC30437

UAS-bsk^{DN}	[9]
UAS-bsk$^{\text{RNAi-GD10555}}$	VDRC34138
UAS-bsk$^{\text{RNAi-TRiP.HMS00777}}$	BL32977
UAS-Camta$^{\text{RNAi-TRiP.HMS02016}}$	BL40849
UAS-CG15514$^{\text{RNAi-TRiP.JF02231}}$	BL31940
UAS-CG18262$^{\text{RNAi-TRiP.HMS00215}}$	BL33348
UAS-CG31441$^{\text{RNAi-GD10260}}$	VDRC21345
UAS-chn$^{\text{RNAi-TRiP.JF02343}}$	BL26779
UAS-dcr2	VDRC60010
UAS-Dll$^{\text{RNAi-TRiP.JF02499}}$	BL29337
UAS-dom$^{\text{RNAi-TRiP.HMC04203}}$	BL55917
UAS-Dsor1$^{\text{RNAi-TRiP.JF03100}}$	BL28685
UAS-Eip93F$^{\text{RNAi-KK108140}}$	VDRC104390
UAS-eyg$^{\text{RNAi-TRiP.JF02124}}$	BL26226
UAS-FLP, tub-GAL80^{ts}*; act>STOP>LHV*$_2$	[10]
UAS-GAL4	DGRC108-491
UAS-grk$^{\text{RNAi-TRiP.HMC04213}}$	BL55926
UAS-Gug$^{\text{RNAi-TRiP.HMS00756}}$	BL32961
UAS-hep$^{\text{RNAi-TRiP.JF03137}}$	BL28710
UAS-Jra$^{\text{RNAi-TRiP.JF01184}}$	BL31595
UAS-kay$^{\text{RNAi-TRiP.HMS00254}}$	BL33379
UAS-lic$^{\text{RNAi-TRiP.HMS05002}}$	BL60010
UAS-lid$^{\text{RNAi-TRiP.HM05155}}$	BL28944
UAS-lilli$^{\text{RNAi-TRiP.HMS01066}}$	BL34592
UAS-lola$^{\text{RNAi-TRiP.JF02254}}$	BL26714
UAS-mCD8:GFP	DGRC108-068
UAS-Mekk1$^{\text{RNAi-TRiP.HM05075}}$	BL28587
UAS-MESR4$^{\text{RNAi-GD11465}}$	VDRC21976
UAS-Nf-YA$^{\text{RNAi-TRiP.JF02013}}$	BL25991
UAS-Nf-YB$^{\text{RNAi-GD4305}}$	VDRC45660
UAS-Nf-YC$^{\text{RNAi-TRiP.HMJ22254}}$	BL58234
UAS-p38a$^{\text{RNAi-TRiP.HMS01224}}$	BL34744
UAS-p38b^{KD}	[11]
UAS-p38c$^{\text{RNAi-TRiP.HMC05719}}$	BL64846
UAS-Pcl$^{\text{RNAi-TRiP.HMS00897}}$	BL33946
UAS-pdm3$^{\text{RNAi-GD4312}}$	VDRC30538
UAS-pnt.P1	BL869
UAS-pnt$^{\text{RNAi-KK100473}}$	VDRC105390
UAS-pnt$^{\text{RNAi-TRiP.JF02227}}$	BL31936
UAS-Psc$^{\text{RNAi-TRiP.HMS01706}}$	BL38261
UAS-Raf$^{\text{RNAi-TRiP.HMC04133}}$	BL55863
UAS-Ras85D$^{\text{RNAi-TRiP.JF02478}}$	BL29319
UAS-rl$^{\text{RNAi-TRiP.HMS00173}}$	BL34855
UAS-sbb$^{\text{RNAi-GD10734}}$	VDRC41845
UAS-schlank$^{\text{RNAi-TRiP.JF02502}}$	BL29340
UAS-Sin3A$^{\text{RNAi-TRiP.HMS00359}}$	BL32368
UAS-spi$^{\text{RNAi-TRiP.JF03322}}$	BL28387
UAS-ss$^{\text{RNAi-GD4491}}$	VDRC10715
UAS-Stat92E$^{\text{RNAi-TRiP.HMS00035}}$	BL33637
UAS-Su(H)$^{\text{RNAi-TRiP.HM05110}}$	BL28900
UAS-svp.II	[12]
UAS-svp1	[13]
UAS-svp$^{\text{RNAi-TRiP.JF03105}}$	BL28689
UAS-sv$^{\text{RNAi-KK102302}}$	VDRC107343
UAS-tara$^{\text{RNAi-GD10757}}$	VDRC34362
UAS-TFAM$^{\text{RNAi-TRiP.JF02307}}$	BL26744
UAS-Utx$^{\text{RNAi-TRiP.HMS00575}}$	BL34076
UAS-vn$^{\text{RNAi-TRiP.HMC04390}}$	BL56950
UAS-woc$^{\text{RNAi-TRiP.JF02398}}$	BL27057
UAS-YL-1$^{\text{RNAi-TRiP.JF02229}}$	BL31938

Supplementary Table 3. Primary and secondary antibodies.

Antibody	Dilution	Source
Rabbit α-Amos	1:500	[14]
Mouse α-Dac	1:20	DSHB, Iowa
Rat α-Elav	1:20	DSHB, Iowa
Chicken α-GFP	1:2000	Abcam (ab13970)
Rabbit α-GFP	1:1000	Invitrogen (A6455)
Mouse nc82	1:10	DSHB, Iowa
Rabbit α-Ato	1:200	[14]
Rabbit α-Sens	1:1000	[15]
Mouse α-Svp	1:10	DSHB, Iowa
Rabbit α-Pon	1:200	[16]
Guinea pig α-Ham	1:30	[17]
Mouse α-Cut	1:10	DSHB, Iowa
Mouse α-Pros	1:10	DSHB, Iowa
Rabbit α-Pnt	1:1000	[18]
Rabbit α-Orco	1:200	[19]
Guinea pig α-Ir8a	1:300	[3]
Rabbit α-Lush	1:100	[20]
α-DIG-POD	1:300	Roche Diagnostics AG (11 207 733 910)
α-FITC-POD	1:300	Roche Diagnostics AG (1 426 346)
α-rabbit Cy3	1:1000	Jackson ImmunoResearch (111-165-144)
α-mouse Cy5	1:250	Jackson ImmunoResearch (115-175-166)
α-chicken Alexa 488	1:1000	Abcam (ab150169)
α-rabbit Alexa 488	1:1000	Invitrogen (A11034)
α-rat Alexa 467	1:250	Jackson ImmunoResearch (112-606-062)
α-rabbit Cy5	1:250	Jackson ImmunoResearch (111-175-144)
α-mouse Cy3	1:1000	Jackson ImmunoResearch (115-165-166)
α-guinea pig Cy3	1:1000	Jackson ImmunoResearch (106-166-003)

Supplementary Table 4. Primers used for generating RNA FISH probes.

Probe	Forward primer	Reverse primer
Gr21a	CCAACCAACGCCAGTTTCTG	CCACCCATAGATGGCGGTAC
Or10a	CCTAATGCTGCGCTTTCGTC	CAGCGCAATTATGGAACCCG
Or42b	ATGGTCTTCGAGCTAATACGTCC	GACTCGCAGTCCTCCATGATGAG
Or67d	GAATTCATGTTGAAAATGGCAAAAGTT	CTCGAGTTATCCCAAATAGTTCATCAA
Or83c	GTCCTTCAAGTCGCTTTCGC	AGAATGGTGCCCAGAATGCA
Or85b	TGAAGCAGATCTGTCATAGTGG	CTACACAGCAGTGGGCATACGG
Or92a	GTGACGATGAAGTCATCACCTTC	CATTTCATCGCCGTAGTAACAG
Or22a	[21]	
Or59b	[21]	
Or85a	[21]	
Ir75b/c	[22]	

Supplementary Table 5. Additional GAL4 drivers tested to identify OSN lineage markers.

**Driver**	**Source**
GMR17C01-GAL4	BL45425
GMR31H11-GAL4	BL45583
GMR17D08-GAL4	BL45818
GMR86F03-GAL4	BL45917
GMR64A03-GAL4	BL46512
GMR70G10-GAL4	BL46640
GMR80G08-GAL4	BL47075
GMR82C05-GAL4	BL40136
GMR18B07-GAL4	BL47476
GMR28E06-GAL4	BL47522
GMR82H03-GAL4	BL47783
GMR31H10-GAL4	BL48104
GMR44F03-GAL4	BL48157
GMR71F08-GAL4	BL48304
GMR71F08-GAL4	BL48754
GMR19C06-GAL4	BL48843
GMR29E03-GAL4	BL49341
GMR38A11-GAL4	BL49980
Dpp-GAL4	BL1553

Supplementary References

1. Li Q*, et al.* A functionally conserved gene regulatory network module governing olfactory neuron diversity. *PLOS Genet.* **12**, e1005780 (2016).

2. Holohan EE, zur Lage PI, Jarman AP. Multiple enhancers contribute to spatial but not temporal complexity in the expression of the proneural gene, *amos*. *BMC Dev. Biol.* **6**, 53 (2006).

3. Abuin L, Bargeton B, Ulbrich MH, Isacoff EY, Kellenberger S, Benton R. Functional architecture of olfactory ionotropic glutamate receptors. *Neuron.* **69**, 44-60 (2011).

4. Lai SL, Lee T. Genetic mosaic with dual binary transcriptional systems in *Drosophila*. *Nat. Neurosci.* **9**, 703-709 (2006).

5. Couto A, Alenius M, Dickson BJ. Molecular, anatomical, and functional organization of the *Drosophila* olfactory system. *Curr. Biol.* **15**, 1535-1547 (2005).

6. Wang JW, Wong AM, Flores J, Vosshall LB, Axel R. Two-photon calcium imaging reveals an odor-evoked map of activity in the fly brain. *Cell.* **112**, 271-282 (2003).

7. Sweeney LB*, et al.* Temporal target restriction of olfactory receptor neurons by Semaphorin-1a/PlexinA-mediated axon-axon interactions. *Neuron.* **53**, 185-200 (2007).

8. Li Q*, et al.* Combinatorial rules of precursor specification underlying olfactory neuron diversity. *Curr. Biol.* **23**, 2481-2490 (2013).

9. Bossuyt W, De Geest N, Aerts S, Leenaerts I, Marynen P, Hassan BA. The atonal proneural transcription factor links differentiation and tumor formation in *Drosophila*. *PLOS Biol.* **7**, e40 (2009).

10. Yagi R, Mayer F, Basler K. Refined LexA transactivators and their use in combination with the *Drosophila* Gal4 system. *Proc. Natl. Acad. Sci. USA.* **107**, 16166-16171 (2010).

11. Terriente-Felix A, Perez L, Bray SJ, Nebreda AR, Milan M. A *Drosophila* model of myeloproliferative neoplasm reveals a feed-forward loop in the JAK pathway mediated by p38 MAPK signalling. *Disease Models & Mech.* **10**, 399-407 (2017).

12. Kerber B, Fellert S, Hoch M. Seven-up, the *Drosophila* homolog of the COUP-TF orphan receptors, controls cell proliferation in the insect kidney. *Genes Dev.* **12**, 1781-1786 (1998).

13. Kramer S, West SR, Hiromi Y. Cell fate control in the *Drosophila* retina by the orphan receptor seven-up: its role in the decisions mediated by the ras signaling pathway. *Development.* **121**, 1361-1372 (1995).

14. zur Lage PI, Prentice DR, Holohan EE, Jarman AP. The *Drosophila* proneural gene *amos* promotes olfactory sensillum formation and suppresses bristle formation. *Development.* **130**, 4683-4693 (2003).

15. Nolo R, Abbott LA, Bellen HJ. Senseless, a Zn finger transcription factor, is necessary and sufficient for sensory organ development in *Drosophila*. *Cell.* **102**, 349-362 (2000).

16. Jia M*, et al.* The structural basis of Miranda-mediated Staufen localization during *Drosophil*a neuroblast asymmetric division. *Nat. Commun.* **6**, 8381 (2015).

17. Moore AW, Jan LY, Jan YN. hamlet, a binary genetic switch between single- and multiple- dendrite neuron morphology. *Science.* **297**, 1355-1358 (2002).

18. Pascual J*, et al.* Hippo reprograms the transcriptional response to Ras signaling. *Dev. Cell.* **42**, 667-680 e664 (2017).

19. Benton R, Sachse S, Michnick SW, Vosshall LB. Atypical membrane topology and heteromeric function of *Drosophila* odorant receptors *in vivo*. *PLOS Biol.* **4**, e20 (2006).

20. Gomez-Diaz C, Reina JH, Cambillau C, Benton R. Ligands for pheromone-sensing neurons are not conformationally activated odorant binding proteins. *PLOS Biology.* **11**, e1001546 (2013).

21. Vosshall LB, Wong AM, Axel R. An olfactory sensory map in the fly brain. *Cell.* **102**, 147-159 (2000).

22. Prieto-Godino LL*, et al.* Evolution of acid-sensing olfactory circuits in drosophilids. *Neuron.* **93**, 661-676 e666 (2017).
