# Supplementary material for: Sensory neuron lineage mapping and manipulation in the Drosophila olfactory system
Source: Nat Commun. 2019 Feb 7;10:643. doi: 10.1038/s41467-019-08345-4 (PMC6367400; doi:10.1038/s41467-019-08345-4)
Supplement: Supplementary file 3 — Description of Additional Supplementary Files [file 41467_2019_8345_MOESM3_ESM.pdf]

Description of Additional Supplementary Files

File Name: Supplementary Data 1

Description: Chemosensory receptor transcript abundance comparison for four paired samples of control and *pnt* RNAi antennae.
